# The Probiotic *Lactobacillus fermentum* Biocenol CCM 7514 Moderates *Campylobacter jejuni*-Induced Body Weight Impairment by Improving Gut Morphometry and Regulating Cecal Cytokine Abundance in Broiler Chickens

**DOI:** 10.3390/ani11010235

**Published:** 2021-01-19

**Authors:** Miroslava Anna Šefcová, Marco Larrea-Álvarez, César Marcelo Larrea-Álvarez, Viera Karaffová, David Ortega-Paredes, Christian Vinueza-Burgos, Zuzana Ševčíková, Mikuláš Levkut, Róbert Herich, Viera Revajová

**Affiliations:** 1 School of Biological Sciences and Engineering, Yachay-Tech University Hacienda San José, Urcuquí-Imbabura 100650, Ecuador; miroslava.sefcova@gmail.com (M.A.Š.); malarrea@yachaytech.edu.ec (M.L.-Á.); 2 Research Unit, Life Science Initiative (LSI), Quito 170102, Ecuador; cmla88@hotmail.com; 3 Department of Morphological Disciplines, University of Veterinary Medicine and Pharmacy, Komenského 73, 040 01 Košice, Slovakia; viera.karaffova@uvlf.sk (V.K.); zuzana.sevcikova@uvlf.sk (Z.Š.); mikulas.levkut@uvlf.sk (M.L.); robert.herich@uvlf.sk (R.H.); 4 Unidad de Investigación en Enfermedades Transmitidas por Alimentos y Resistencia a los Antimicrobianos (UNIETAR), Facultad de Medicina Veterinaria y Zootecnia, Universidad Central del Ecuador, Quito 170129, Ecuador; daortegap@gmail.com (D.O.-P.); cvinueza@uce.edu.ec (C.V.-B.); 5 Institute of Neuroimmunology, Slovak Academy of Science, Dúbravská cesta 5779/9, 845 10 Bratislava, Slovakia

**Keywords:** *Lactobacillus fermentum*, *Campylobacter jejuni*, broiler chicken, body weight, crypt depth, small intestine, villus height, cytokine response, IL-1β, IL-18, IL-17, IL-15, IL13, IL-4

## Abstract

**Simple Summary:**

High consumption of chicken meat and derived products has been associated with *Campylobacter jejuni* infections in humans. Probiotics have been exploited successfully with the aim of preventing colonization by unwanted microorganisms in birds. In this research, we investigated the effects of *Lactobacillus fermentum* Biocenol CCM 7514 supplementation on body weight, morphometry of the intestine and the cecal cytokine response. Probiotic-treated chickens showed higher body weight values than those exposed to *C. jejuni* or reared under control conditions. These differences in body weight were correlated to the overall characteristics of the small intestine, with larger villi and deeper crypts, observed in chickens administered with *L. fermentum*; such conditions are known to favor nutrient absorption. Likewise, body weight proved to be correlated to transcript abundance of IL-1β and IL-13. In probiotic-treated birds, such factors were upregulated in comparison to what was detected in *C. jejuni*-infected chickens; these interleukins are considered crucial in the response to invading pathogens. Clearly, these results show that administration of this probiotic strain lessens the negative effects elicited by *C. jejuni* and ultimately improves chicken body weight.

**Abstract:**

This research was conducted to investigate if the administration of the probiotic *Lactobacillus fermentum* could influence body weight, intestinal morphometry and the cecal cytokine response in *Campylobacter jejuni*-infected chickens. Seventy-two 1-day old COBB 500 male chicks were allocated randomly into four experimental groups. (I) Control group (C), in which chicks were left untreated. (II) LB group, treated with *L. fermentum*. (III) Cj group, infected with *C. jejuni* and (IV) coexposure group in which both bacteria were administered. Body weight was registered and then all birds were slaughtered; samples from the small intestine and caecum were collected at 4- and 7-days post infection. The experiment lasted eleven days. Villi height and crypt depth ratios of the duodenum, jejunum and ileum were evaluated using appropriate software, while reverse transcription quantitative PCR (RT-qPCR) was utilized for assessing transcript levels of key cecal inflammatory cytokines (IL-1β, IL-18, IL-17, IL-15, IL13 and IL-4). *Campylobacter*-infected birds showed lower body weight values than those supplemented with the probiotic; these birds, in turn, proved to be heavier than those reared under control conditions. *L. fermentum* administration improved morphometrical parameters of the duodenum, jejunum and ileum; in general, villi were larger and crypts deeper than those identified in control conditions. Moreover, the negative effects elicited by *C. jejuni* were not observed in chickens exposed to the probiotic. Significant differences were also determined with regards to transcript abundance of all evaluated cytokines in the caecum. *C. jejuni* induced a downregulation of the studied interleukins; however, such a response was heightened by administration of *L. fermentum*, with an increase rate of transcription that promoted a more effective response to a *C. jejuni* infection. The effects of experimental treatments proved to vary between sampling points. Conclusively, these results demonstrate that *L. fermentum* lessens the negative effects elicited by *C. jejuni* on body weight by alleviating the impact on intestinal morphometry and cecal cytokine response, which ultimately improve chicken growth performance.

## 1. Introduction

The digestive tract serves as a selective regulator of nutrient intake and is the site of interaction with commensal and pathogenic bacteria [1]. Pathogenic colonization of the gut has negative effects on enterocyte permeability, ion transport and the structure of the mucosa [2]. *Campylobacter jejuni* is considered responsible for several gastrointestinal diseases in humans, with high consumption of chicken meat and poultry products being associated with human infections [3,4].

*C. jejuni* is capable of colonizing the avian gut in high concentrations and, upon interaction with epithelial or dendritic cells, stimulates production of proinflammatory cytokines including IL-1, TNF-α, IFN-γ, IL-2 and IL-6 [5]. *C. jejuni*-associated molecular patterns (e.g., lipooligosaccharide—LOS) are able to activate particular Toll-like receptors (TLRs) that stimulate downstream signaling and promote cytokine release [6]. However, it has been reported that this initial response is later downregulated, as the host immune reaction seems to reach a level of tolerance [7,8]. This reflects the fact that the chicken immune response is inefficiently activated by *C. jejuni* [9], which may contribute to the pathological effects exerted by these bacteria. In particular, fast-growing broiler breeds, such as the extensively commercialized COBB 500, are known to be susceptible to a *C. jejuni* infection [10,11,12]. In particular, it has been observed that *C. jejuni* is capable of affecting villus and crypt metrics in the small intestine, this would certainly decrease the absorption surface for nutrient uptake leading, thus, to a decrease of feed utilization [5,13]. These conditions can affect growth performance, induce damage of the intestinal mucosa and cause neurological-related conditions, which may have an impact in poultry production [14].

Evidently, antibiotics have been used intensively in farms as a way to counteract the effects of Campylobacteriosis, notwithstanding the associated selection of resistant bacterial strains [15,16,17]. Recently, the concept of early life programming has gained significant attention, as it assumes that environmental exposure during critical pre- or early post-natal periods can influence the development of diseases later in life. It appears particularly relevant for broiler breeds selected for rapid growth in which the immune system must develop during early phases [18]. For example, exposure to probiotic *Lactobacilli* (*Lactobacillus ingluviei*, *L. agilis* and *L. reuteri*), immediately post-hatching, not only increased body weight in chickens by 28 days of age, but also induced a reduction of pathogenic bacteria including *Shigella* and *Escherichia* species [19].

Probiotics have been employed, with successful results, to prevent colonization by pathogenic bacteria [20]. Particularly, various *Lactobacillus* strains have proved advantageous for lessening the magnitude of a *C. jejuni* infection [21]. As mentioned previously, early infection of the gut (4 days of age) by these bacteria elicits a marked initial immune response that entails the regulation of various cytokines; such a reaction could be modulated by early supplementation of probiotics [10,11,22]. Recently, we demonstrated that early supplementation of *L. fermentum* Biocenol CCM 7514 was capable of upregulating the immune response in broilers challenged with *C. coli* by 8 days of age; a higher percentage of immunoglobulins and CD8 cells were detected, which proved to be correlated to the increased transcription of Th2 cytokines, including IL-4 and IL-13 [23]. Moreover, we have also reported that administration of this probiotic strain, during the first week of development, influences positively the expression proinflammatory cytokines (IL-15, IL-1β, IL-17 and IL-18) and reduces the negative effects, with regards to body weight, observed in broilers infected with *C. jejuni* by 5 days of age [11]. Therefore, in this research, we sought to investigate the effects of *L. fermentum* supplementation on body weight in *C. jejuni*-challenged chickens at 4- and 7-days post-infection. Additionally, we aimed at assessing potential associations between such effects and the influence of the probiotic on small intestinal morphometry and cecal transcription of the aforementioned cytokines.

## 2. Materials and Methods

### 2.1. Chickens and Experimental Scheme

Seventy-two 1-day-old COBB 500 male cock chicks were used in this investigation with constant access to water and feed ad libitum; the diet did not contain any antibiotics, probiotics or coccidiostats. The following experimental groups were included in the study: (I) control, in which chickens were not challenged with any bacteria (C, *n* = 18), (II) probiotic group, where birds were inoculated with *L. fermentum* Biocenol CCM 7514 (LB, *n* = 18), (III) *Campylobacter* group, in which birds were infected with *C. jejuni* (Cj, *n* = 18), and (IV) coexposure group, in which both bacteria were administered (LBCj, *n* = 18); the experimental design was based on previous research [11,23]. Per group, birds were split into two equally separated subgroups, as sampling was carried out at two different time points (Table 1). At each time point, three birds were weighed, sacrificed and sections of the small intestine and caecum were sampled, this process was performed in triplicate (*n* = 9). Birds were raised on the floor (9 birds/m^2^) with a temperature between 29 and 32 °C throughout the investigation, which lasted 11 days. During the first two days, chickens were exposed to a regime of 24 h of continuous light; subsequently the regime changed to a one of 23 h of light and one of dark. Environmental conditions were kept in accordance with broiler breeding criteria [24].

Bacterial strains of *L. fermentum* Biocenol CCM 7514 and *C. jejuni* CCM 6189 were grown as previously detailed [11,23]. A suspension of *L. fermentum* (10^9^ colony-forming units—CFU—/0.2 mL) was supplemented, per os, daily for one week to groups LB and LBCj. On day 4, a dose of 10^8^ CFU/0.2 mL of *C. jejuni* was supplemented, per os, to chickens from groups Cj and LBCj. Samples were collected on day 8 (4 days post-infection—dpi), and on day 11 (7 dpi) (Table 1). Sections from the duodenum, jejunum and ileum were prepared for morphometrical analyses, while sections from the caecum were kept in RNA-later (Thermo Scientific, Waltham, MA, USA) and stored at –80 °C. All animal work was carried out according to the guidelines for the care and use of experimental animals recognized by the Ethical Commission of the University of Veterinary Medicine and Pharmacy in Košice and was accepted by the Slovak Republic National Veterinary and Food Administration, protocol no. 863/17-221.

### 2.2. Body Weight of Chickens 

An analytical scale (BOECO, Hamburg, Germany) was used for weighing birds quotidianly and on day 8 (4 dpi) and 11 (7 dpi) (Table 1). 

### 2.3. Morphometrical Analyses

Duodenum, jejunum and ileum sections (2 cm) were fixed using a 10% formalin solution for 2 days, and later dehydrated by consecutive washes with ethyl alcohol (70–100%). Subsequently, xylol was used to diaphanize the samples, which were then inserted in paraffin blocks. A microtome was used to cut such blocks in three longitudinal sections of 5 μm thick blades stained by hematoxylin–eosin. Morphometry of sections was evaluated using image capture, while villus height and crypt depth was assessed with NIS-Elements Advanced Research 3.0 (Nikon, Tokyo, Japan). Per segment, villus height and crypt depth were individually assessed on different intact villi (seven at least). The presence of an intact lamina propria was used as a benchmark for villus choice. The villus height to crypt depth ratio was calculated by dividing the height of villi by the depth of crypts [25].

### 2.4. RNA Extraction and RT-qPCR Assays 

RNA purification was carried out employing the RNeasy mini kit (Qiagen, Hilden, Germany) following the provided guidelines. The iScript cDNA Synthesis Kit (Bio-Rad, Hercules, CA, USA) was used for reverse transcription, the cDNA obtained was diluted in 10× in UltraPure™ DNase/RNase-Free distilled water (Invitrogen, Waltham, MA, USA) and stored at –80 °C for further use. Primers utilized herein are enumerated in Table S1. Cycling conditions, detection, amplification, assessment of melting curve and normalization of data were arranged as hitherto described [26]. The Ct values were normalized to a Ct value of a reference gene (GAPDH) (Delta—Δ—Ct), and calculated as 2^−ΔCt^. Samples were tested twice and means were utilized for further calculations. 

### 2.5. Statistical Analyes 

A principal component analysis (PCA) was chosen as a tool for data exploration. Significant differences among the experimental groups were assessed using ANOVA, along with the Tukey post hoc test, while differences between sampling points were assessed using Student’s *t*-test. Pearson’s *r* correlation coefficient was employed to assess the relationships between the indicators. Analyses were carried out using MATLAB^®^ 9.9.9341360 (R2016a) (MathWorks, Natick, MA, USA), and figures were developed with Python’s plotting library, Matplotlib 3.0.3 (Python Software Foundation, Fredericksburg, VA, USA).

## 3. Results

### 3.1. Chickens’ Body Weight 

**Figure 1:**
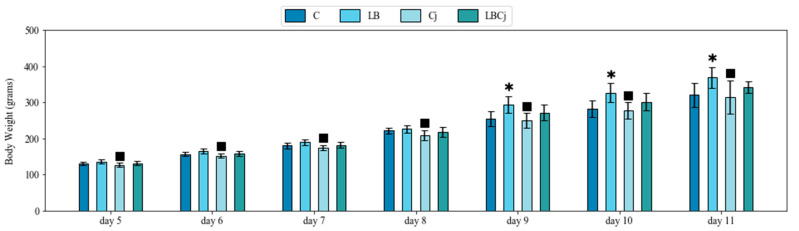
Effects on body weight of *L. fermentum* and *C. jejuni* colonization at two-time points. Bodyweight average values of treated and untreated animals from day 5 (1 dpi) to 11 (7 dpi). Values are mean ± SE (*n* = 9). * designates significant differences with the control group; ^■^ with the probiotic group. C, control; LB, *L. fermentum*; Cj, *C. jejuni*; LBCj, coexposure; dpi, days post-infection; SE, standard error.

No significant differences were observed with regards to body weight during the first 4 days of the experiment (Table S2). From day 5 (1 dpi) onwards, birds belonging to the LB group proved to be heavier than those infected with *C. jejuni* (*p* < 0.05). Chickens from the control group did not show differences with those of the Cj group, however their body weight, from day 9 (5 dpi) to 11 (7 dpi), was significantly lower (*p* < 0.05) than in those treated with the probiotic. Importantly, the reduction in body weight, detected in the Cj group was not observed in individuals belonging to the coexposure group. Namely, previous administration of *L. fermentum* seemed to prevent the negative effects, elicited by *C. jejuni*, on chicken body weight (Figure 1).

### 3.2. Morphometrical Analyses

**Figure 2:**
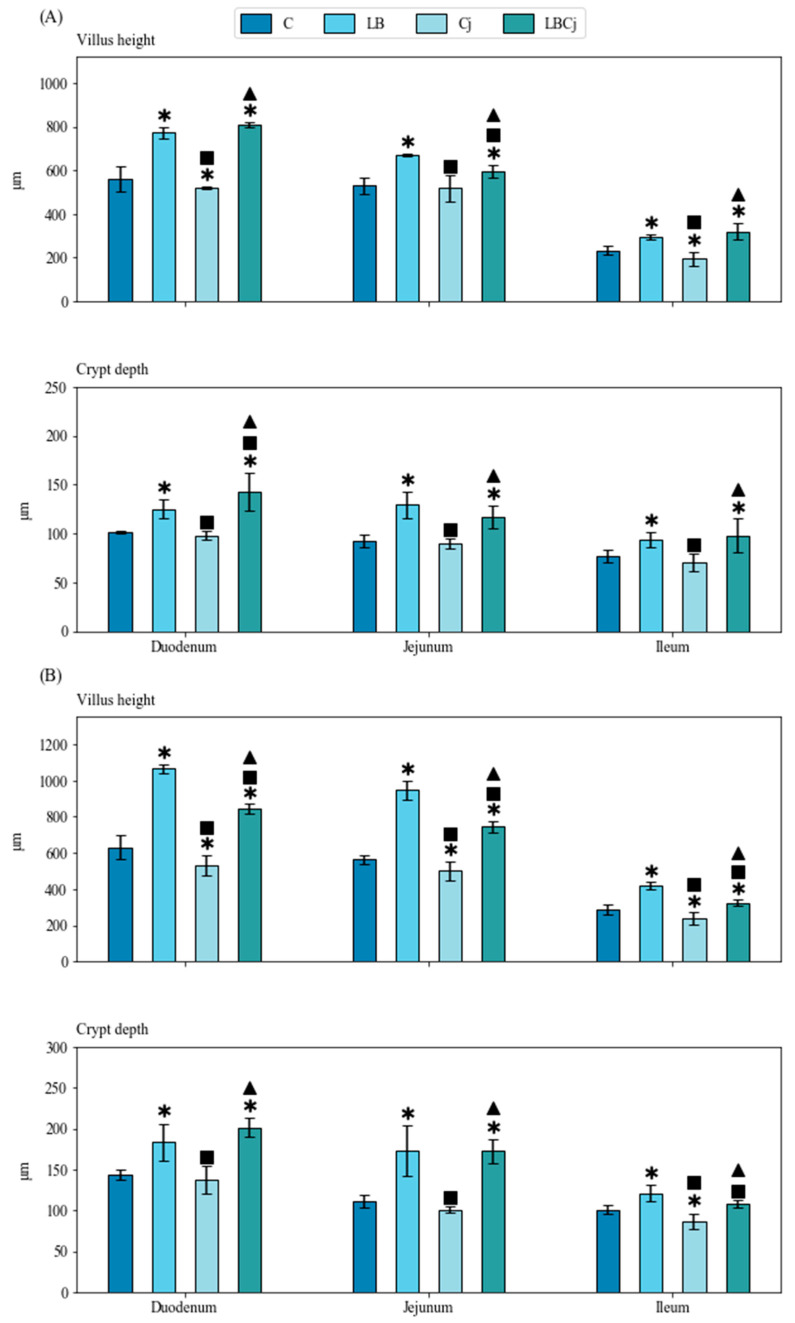
Effects of bacterial treatment on intestinal morphometry at two sampling points. (**A**) Four days post-infection (dpi) and (**B**) 7 dpi. Values are mean ± SE (*n* = 9). * designates significant differences with the control group; ^■^ with the probiotic group; ^▲^ with the *C. jejuni* group. C, control; LB, *L. fermentum*; Cj, *C. jejuni*; LBCj, coexposure; dpi, days post-infection; SE, standard error.

Results of intestinal morphometry are shown in Figure 2. At the first time point (4 dpi), supplementation of the probiotic augmented the height of villi and the depth of crypts in the duodenum, jejunum and ileum compared to control conditions. *C. jejuni* treatment, on the other hand, did not modify significantly the morphometry of such sections compared to untreated birds, notwithstanding the reduction observed in villus height of the ileum. Infection with the pathogen induced a decrease in average values of villi height and crypt depth compared to probiotic-exposed birds. This negative influence was not observed in the coexposure group, in which the average values were similar to those observed in the probiotic treatment (Figure 2A).

A similar effect was observed at 7 dpi, with *L. fermentum* administration increasing significantly villus height and crypt depth of all sections, compared to control conditions and *C. jejuni* inoculation. Chickens infected with *C. jejuni* showed lower average values of villi height in the duodenum, jejunum and ileum than untreated birds; ileum crypt depth was also lower in pathogen-treated birds than in those belonging to the other groups. Arguably, the negative effects induced by *C. jejuni* exposure were alleviated by the presence of the probiotic. Values of crypt depth incremented significantly between time points, except for the ileum of chickens belonging to the coexposure group. The height of villi increased between sampling points in probiotic treated birds (Figure 2B). At both time points, significant differences were also revealed in the villus height to the crypt depth ratio in the duodenum between *C. jejuni* and *L. fermentum* exposed birds, with probiotic treated chickens showing in general higher values. At 7 dpi, the LB group yielded higher values than the control and Cj groups in both the duodenum and ileum (Table 2).

### 3.3. Cytokine Response

**Figure 3:**
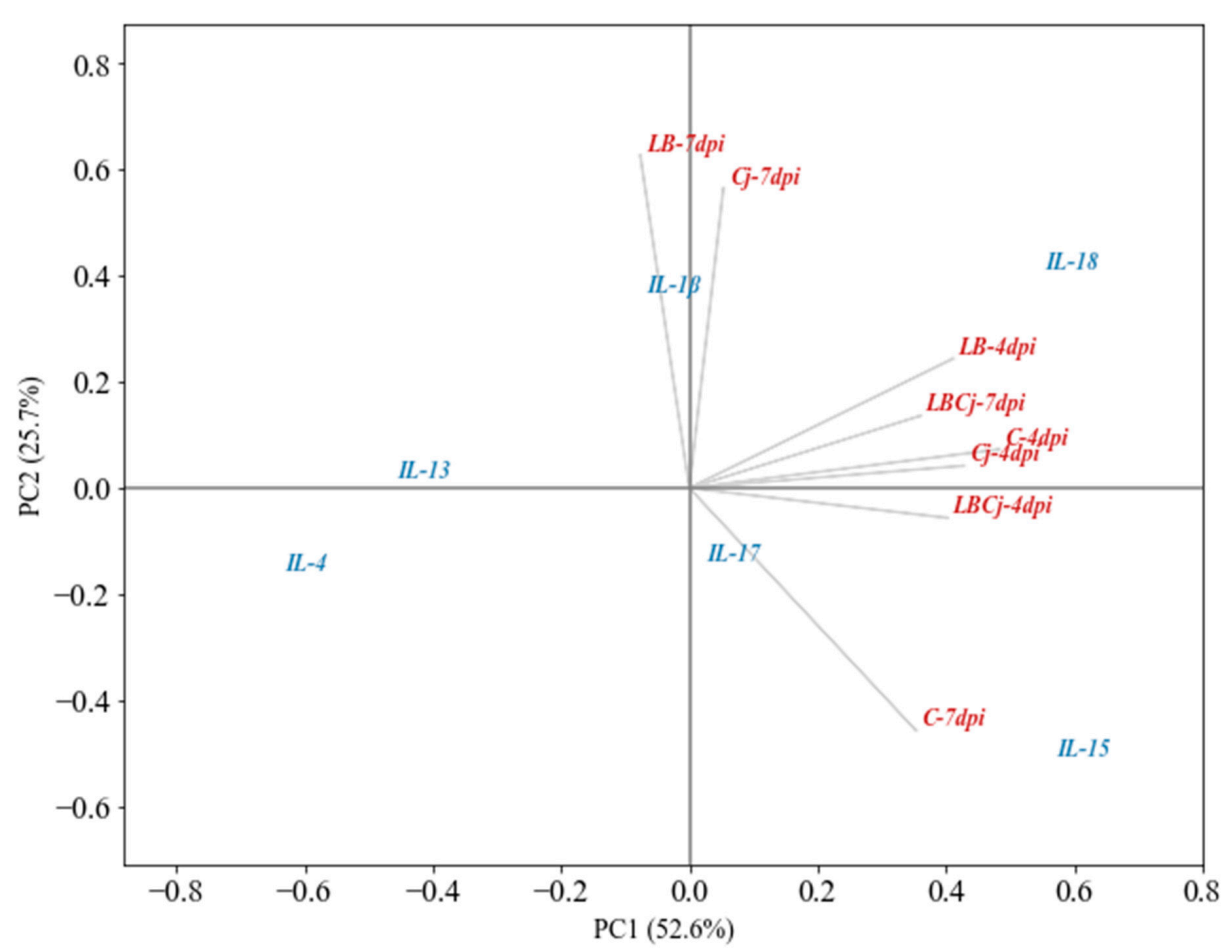
Ordination map by principal component analysis (PCA) of the cecal cytokine response, at 4 days post-infection (dpi) and 7 dpi, in birds exposed to *L. fermentum* and *C. jejuni*.

**Figure 4:**
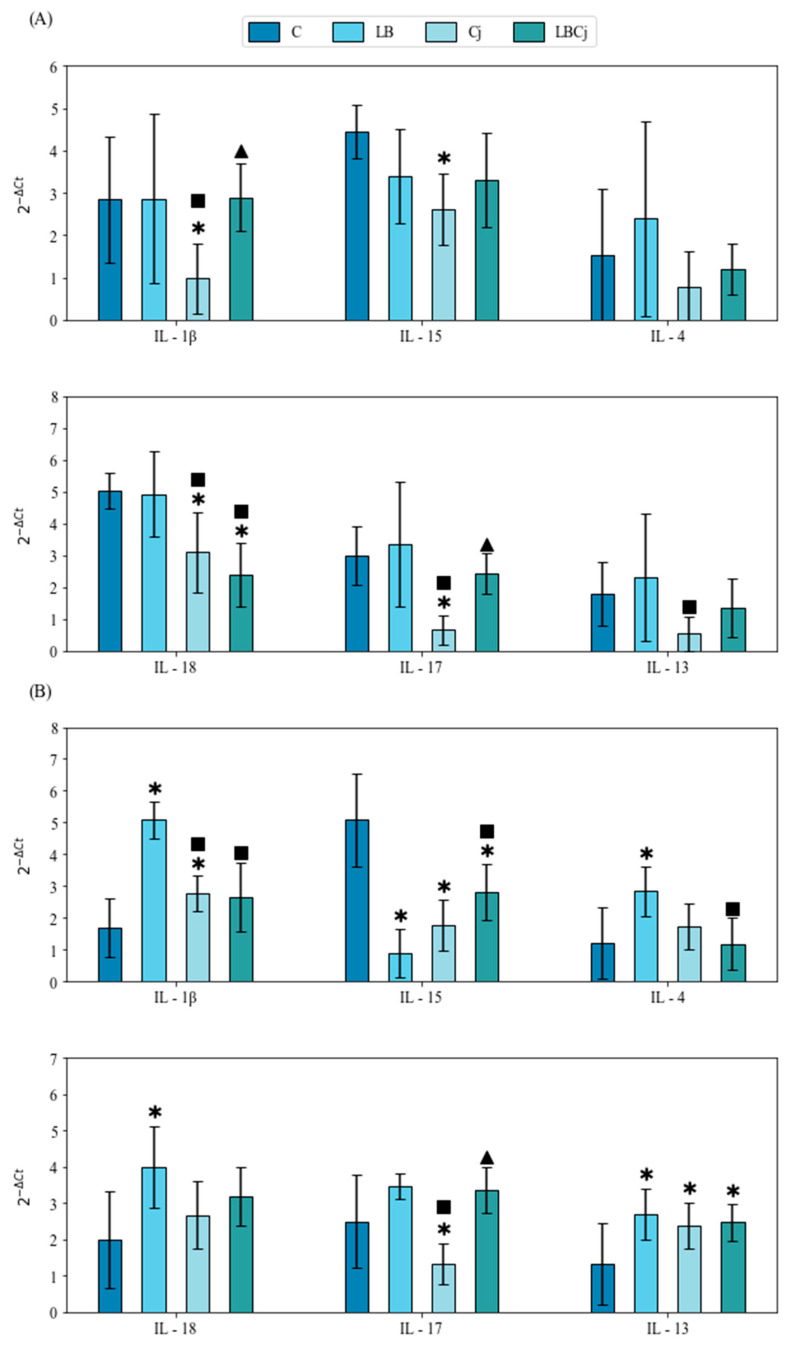
Effects of bacterial supplementation on cecal cytokine transcript abundance. (**A**) Four days post-infection (dpi) and (**B**) 7 dpi. Values are mean ± SE (*n* = 9). * designates significant differences with the control group; ^■^ with the probiotic group; ^▲^ with the *C. jejuni* group. Different superscript letters show significant differences between time points of each experimental group. C, control; LB, *L. fermentum*; Cj, *C. jejuni*; LBCj, coexposure; SE, standard error.

PCA was used to explore the variation of cecal cytokines (mRNA levels) in response to the administered bacteria (Figure 3). The axes identified (PC1 and PC2), called principal components, correspond to the sources of greatest variance. PCA is a method of data reduction that calculate a few independent, uncorrelated factors (axes) that represent linear combinations of the variables explaining most of the variation in the data [27]. Figure 3 shows that the first two components explain 78.3% of the variation with PC1 contributing 52.6% and PC2 25.7%. The results show that transcript response in the groups, located on the right-hand side of the graph, explained most of the variation for component 1, whereas the experimental groups LB, Cj and control (at 7 dpi) did so for component 2. The cytokine whose transcript abundance varied the most was IL-15 followed by IL-18, IL-17, IL-1β, IL-13 and IL-4. At 4 dpi, all experimental groups displayed a similar pattern of cytokine transcript production. IL-18 and IL-15 were the most abundant followed by IL-17 and IL-1β, while IL-13 and IL-4 were the least. At 7 dpi, this pattern of expression was maintained, notwithstanding the upregulation of IL-1β in the LB and Cj groups (Figure 3). Significant differences in mRNA abundance were mainly observed in *C. jejuni*-treated chickens. In these birds, expression of the evaluated inflammatory mediators (IL-1β, IL-15, IL-18 and IL-17) was downregulated compared to untreated and *L. fermentum*-exposed chickens, whereas expression of Th2 cytokines was not modified, except for a reduction of IL-13 in chicks infected with *C. jejuni* compared to those supplemented with the probiotic (Figure 4A).

Three days later, at 7 dpi, this pattern changed in favor of a higher production of IL-1β in groups treated with the probiotic and the pathogen separately; in the control group, IL-15 and IL-17 were the most abundant at this stage (Figure 3). Indeed, administration of both bacteria, either alone or in combination, reduced significantly the expression of IL-15, while downregulation of IL-17 was detected uniquely after infection with *C. jejuni*. Between sampling points, abundance of IL-15 was lowered significantly (3.8-fold) after probiotic supplementation, while IL-17 was upregulated (2-fold) in pathogen-treated birds and in those belonging to the coexposure group (1.4-fold) (Figure 4B). At 7 dpi, IL-1β and IL-18 were significantly upregulated compared to control conditions by treatment with *L. fermentum*; levels of the IL-18 did not vary between sampling points in the experimental groups, although its abundance in the control group was significantly reduced (2.5-fold). Contrarily, production of IL-1β was augmented 1.8 times from day 8 (4 dpi) to 11 (7 dpi) in chicks exposed to the probiotic and 2.8 times in those infected with *C. jejuni* (Figure 4B). Transcription of Th2 cytokines was significantly higher in birds supplemented with the probiotic compared to those of the control and coexposure groups, although abundance of IL-13 augmented after any bacterial treatment. Levels of IL-4 and IL-13 increased (2.2 times and 4.5 times, respectively) from the 8th to the 11th day in chicks inoculated with *C. jejuni*, albeit IL-13 was also upregulated (1.9-fold) upon exposure to both bacteria (Figure 4B).

### 3.4. Correlation Analysis between Evaluated Indicators

Pearson’s *r* correlation coefficient analysis revealed positive correlations (*p* < 0.05) not only between body weight and villus height of duodenum (0.982), jejunum (0.992) and ileum (0.963), but also between body weight and the response of IL-13 (0.995) and IL-1β (0.996). This means that the increment observed in the height of villi and abundance of such cytokines was directly associated with the body weight gain observed in probiotic-treated birds.

## 4. Discussion

This study aimed at testing the feasibility of post-natal *L. fermentum* administration as a mean to reduce the negative impact of early *C. jejuni* colonization in terms of body weight, intestinal morphometry and cecal cytokine response. Body weight loss has been associated with pathogen-triggered inflammation processes [28]. In particular, infection with *C. jejuni*, at early stages of growth, elicits an important reduction in growth rate and an increased inflammatory response [5]. On the other hand, treatment with *Lactobacillus* has prompted a positive response regarding chicken body weight [29]. For instance, 1-week-old birds exposed to *L. fermentum* evidenced higher body weight values, than those infected with *C. jejuni* by 5 days of age [11]. Similarly, the present results show that supplementation of these probiotic bacteria increased body weight, compared to *C. jejuni* exposure, at even later stages (11 days-of-age). The observed reduction in body weight was not registered in the LBCj treatment, which implies that *L. fermentum* was capable of lessening the negative effects of *C. jejuni* on this physiological parameter. Moreover, and in accordance with previous research, administration of the probiotic induced an overall increment in chicken body weight compared to control conditions, this has also been observed when supplementing other *Lactobacillus* species such as *L. ingluviei*, *L. agilis*, *L. reuteri* and *L. acidophilus* [19,30]. The positive effects revealed in probiotic-treated birds were sustained by the results obtained from both morphometric analyses of the small intestine, and from cytokine transcriptomic analyses of the caecum.

Various key elements could be recognized within the gut ecosystem; among them, intestinal epithelial cells and the immune system are of prime importance. These elements could be modulated by factors such as diet, gender, housing conditions and age of birds [31,32]. Probiotic bacteria, including *Bacillus*, *Enterococcus* or *Lactobacillus* strains, have proved to be beneficial for maintaining the integrity of intestinal epithelial cells [33,34,35,36]; this leads to a superior absorption of nutrients, which ultimately enhances chicken growth performance [30,37]. The present outcomes showed that supplementation of *L. fermentum*, during the first week of growth, improved morphological parameters in the duodenum, jejunum and ileum, with an overall increase in the average values of villus height, crypt depth and villus height-to-crypt depth ratio. On other hand, *C. jejuni* infection induced a reduction of such parameters, especially at 7 dpi. In these birds, villi height and crypt depth tended to be shorter and narrower, respectively, than in those grown under control conditions or exposed to the probiotic. These pathological effects have been reported previously in birds of similar age in which infection proceeded early in their development, however no significant differences were observed at later stages (30 days-of-age) [5].

An increment in crypt depth has been associated with a rapid renewal of the villi [38], which positively influences their height and so increases the surface area capable of nutrient absorption [39]. Likewise, a higher ratio of villus height to crypt has been linked to a greater capacity of nutrient absorption in birds [40]. In this study, *L. fermentum*-treated birds showed higher ratios than those exposed to *C. jejuni* or reared under control conditions; this suggests a heightened proliferative activity in the mucosa, triggered by the probiotic, which ultimately may improve feed efficiency. Microorganisms secrete a variety of molecules and fermented products that modulate migration, proliferation number and apoptosis of intestinal cells. For instance, probiotic supplementation proved to accelerate intestinal enterocyte movement along the crypt-villus axis by activating particular integrin collagen receptors. Additionally, it has been reported that probiotic bacteria can increase the production of short-chain fatty acids, which have been associated with proliferation of intestinal epithelial cells [41]. The outcomes of the present research are in line with previous studies showing the positive effects of *Lactobacillus* supplementation on mucosal architecture in pathogen-exposed birds [42,43], although these studies have also made emphasis on goblet cell hyperplasia and mucin production, which contribute as well for maintaining the integrity of the intestinal barrier. Arguably, our data revealed the importance of *L. fermentum* for preserving the intestinal ecosystem and, most importantly, for preventing epithelial damage induced by *C. jejuni*.

In chickens, the immune reaction to *Campylobacter* spp. is complex, with bacterial detection mediated by pattern recognition receptors (PRR), which after stimulation stimulate synthesis of inflammatory cytokines [44]. In the caecum, which represents a reservoir for *Campylobacter* spp., transcription of inflammatory mediators (IL-1β, IL-15, IL-18 and IL-17) was markedly modified by all bacterial treatments, both at 4 and 7 dpi; whereas transcription of factors promoting the activation and maintenance of the humoral or antibody-mediated response (IL-4 and IL-13) was altered mainly at 7 dpi. Overall, *C. jejuni* elicited the downregulation of inflammatory cytokines. This has been previously reported as there appears to be a decline of the initial response to a *C. jejuni* colonization [7]; initially, the immune system reacts to such bacterial exposure as an attack, albeit it later develops some tolerance [45,46]. In fact, this study showed that levels of inflammatory cytokines, compared to control conditions, were actually reduced by the presence of *C. jejuni*. However, in infected chickens treated with the probiotic, the abundance of these cytokines proved to be similar to that observed in untreated birds. Most likely, the presence of *L. fermentum*, allowed chickens to bypass the negative influence, elicited by *C. jejuni*, with regards to cytokine inflammatory response. Indeed, it has been demonstrated that *L. fermentum* supplementation modified inflammatory cytokine expression in *C. jejuni*-challenged chickens at very early stages of infection, this ultimately helped in relieving the consequences elicited by the pathogen [11]. A variety of receptors in epithelial, dendritic cells or macrophages are activated by molecules such as lipoteichoic acid, (LTA), wall teichoic acid or peptidoglycans, also known as microorganism-associated molecular patterns. *Lactobacillus*-associated patterns are capable of stimulating receptors such as Toll-like receptor 2 (TLR2)–CD1 and/or TLR2–TLR6 heterodimers, this activation promotes downstream signaling and ultimately cytokine expression [47].

IL-1β is principally synthesized by activated macrophages, and is known to be involved in chronic conditions; in particular, elevated levels of this factor have been detected in the inflammatory/immune response mounted by chickens against biotic and abiotic stress [48,49]. Transcription of this interleukin was not altered during the first hours after infection with *C. jejuni*, even in birds previously treated with *L. fermentum*. Apparently, IL-1β did not play a significant role in the response displayed against the presence of these bacteria [11]. However, our data demonstrated that, at later stages of infection, IL-1β was indeed downregulated by *C. jejuni*, whereas supplementation of *L. fermentum* increased its abundance significantly at 7 dpi. Arguably, treatment with this probiotic promotes expression of such a factor, which itself might prompt activation of caspase-1 with subsequent cleavage of pro-IL-1β and pro-IL-18 precursors into active forms [50], this would definitively help birds mount a stronger inflammatory response against *C. jejuni*. Moreover, these data suggest that the probiotic strain used herein might be capable of stimulating NLRP3 inflammasome, as has been demonstrated by strains of *L. reuteri* and *L. rhamnosus* [10,51].

Exposure to *C. jejuni*, with or without the probiotic, induced a downregulation of IL-18 mRNA synthesis at 4 dpi, however supplementation of *L. fermentum* increased the abundance of this factor at 7 dpi. IL-18 is principally synthesized by macrophages, and is known for stimulating the development of T helper cells and the synthesis of IFN-γ by NK cells, CD8 and CD4 T cells [52]. This factor has been identified as an important player in the response to a *C. jejuni* infection mediated by probiotic treatment [21]. At very early stages of infection (12–48 h post infection) IL-18 transcription levels were not altered in birds challenged with *C. jejuni* when supplemented with *L. fermentum* during the first week of growth [11]. However, our results demonstrate upregulation of this interleukin by *L. fermentum* administration, at later stages of infection nonetheless (7 dpi). It proves the ability of this probiotic to trigger similar effects as other Lactobacilli species, including *L. reuteri* or *L. salivarius*, to upregulate IL-18 expression [53]. 

IL-17 and IL-15 transcription was significantly reduced by inoculation with *C. jejuni*, regardless of the sampling point. Probiotic treatment prompted a reaction of IL-17 similar to that observed in untreated birds, whereas synthesis of the IL-15 was downregulated by *L. fermentum* supplementation uniquely at 7 dpi. T helper 17 cells (Th17) synthesized IL-17, which drives the production of a diversity of chemokines that function as attractors of monocytes and neutrophils. This factor has been associated with the pathogenesis of bacterial and parasitic infections [54]. In chickens, an upregulation of IL-17 has been observed as a response to *C. jejuni* colonization [7], especially at very early stages of infection (12 hpi), although levels of this factor proved to be no different to those of untreated or *L. fermentum*-exposed birds in later sampling points (36–48 hpi) [23]. Our results show that transcription of IL-17 was downregulated by the presence of *C. jejuni*, but administration of the probiotic resulted in levels similar to those detected in the control group. Likewise, IL-15 was downregulated by *C. jejuni* administration, similar effects have been previously described, although an upregulation was observed at very early stages of infection (36–48 hpi) [11]. However, our data show that supplementation *L. fermentum* maintained transcript abundance of IL-15 in levels comparable to those found in control conditions. This factor plays the role of an inflammatory mediator capable of T cell activation and proliferation and stimulation of NK cells [55]; this interleukin proved to be an important player in the reaction to a *C. coli* infection in *L. fermentum* exposed birds. IL-15 levels in the caecum were indeed correlated with a higher percentage of CD8 intraepithelial lymphocytes (IELs) in probiotic treated birds than in pathogen-exposed or untreated birds [23]. Clearly, probiotic treatment promoted the transcription of these factors, which would have been otherwise damped by *C. jejuni* invasion. This might result in enhanced recruitment of monocytes and neutrophils and the proliferation of CD8 IELs that eventually could contribute to containment and clearance of unwanted microorganisms, thus improving the animal well-being by avoiding pathogen-elicited responses.

Transcription of the evaluated Th2 cytokines was not markedly modified by any treatment. Upregulation of IL-4 was elicited only by *L. fermentum*, while IL-13 abundance increased in all treatments at 7 dpi, notwithstanding the downregulation observed at 4 dpi by *C. jejuni*. These factors are recognized as important modulators of inflammatory mechanisms and activators of humoral immunity. Actually, these cytokines are known to induce positively the production of both IgM and IgA [56]. In broilers infected with *C. coli*, in the presence of *L. fermentum*, an upregulation of IL-4 and IL-13 was detected in the caecum; this ultimately proved to be correlated with the percentage of IgM and IgA found therein, which was significantly higher than that recorded in untreated and *C. coli*-challenged birds [23]. Certainly, the transcription rate of IL-4 and IL-13, prompted by the probiotic, might stimulate the synthesis of the aforementioned immunoglobulins that are critical for protection the gut mucosa; they act, mainly, by neutralizing or preventing toxins, viruses, or bacteria from binding. Furthermore, immunoglobulins are capable of promoting pathogen phagocytosis and opsonization, which involves bacterial agglutination and complement activation [57]. These effects could undoubtedly help reduce the negative impact of chicken Campylobacteriosis.

Pearson’s *r* coefficient analysis showed positive correlations between body weight and villus height of the duodenum, jejunum and ileum, this means that the increment in body weight was related to the improved architecture of the small intestine induced by probiotic administration. This corroborates previous findings that have associated probiotic treatment to such conditions leading to enhanced nutrient absorption [30,37,39]. Likewise, body weight was positively correlated with transcript production of IL-1β and IL-13. These factors are critical for mounting a more effective reaction against *C. jejuni*. However, birds seemed unable to react promptly to the pathogen unless treated with *L. fermentum*. Probiotic supplementation upregulated transcription of such cytokines, which could have led to a robust defense response. For instance, IL-13, along with IL-4, has been associated with strong antibody production, which might be able to neutralize unwanted bacteria and prevent their attachment to the mucosa [58]. IL-1β, on the other hand, is a potent proinflammatory cytokine that induces neutrophil activation and influx and T cell activation and cytokine production [52]. Undoubtedly, these effects might improve the clearance of pathogenic bacteria that would otherwise induce cecal lesions inducing an overall body weight reduction [59].

## 5. Conclusions

In chickens, probiotics have been widely used as means for reducing the negative effects elicited by unwanted microorganisms in the gut. In this study, we demonstrated that *L. fermentum* Biocenol CCM 7514 was capable of ameliorating intestinal architecture, with deeper crypts and larger villi in the duodenum, jejunum and ileum of chickens challenged with *C. jejuni*. Furthermore, *L. fermentum* stimulated the expression of key Th1 and Th2 cytokines, otherwise downregulated by the pathogen, which could result in a more effective reaction towards a *C. jejuni* invasion. In conclusion, administration of probiotic Lactobacilli elicits a positive effect on morphometric parameters of the small intestine and on components of the cecal inflammatory response, which ultimately seemed to improve broiler growth performance.

## Figures and Tables

**Table 1:** Scheme aimed at assessing body weight, intestinal morphometry and cytokine transcript response in chickens supplemented with the probiotic and *C. jejuni*.

Day	Control (Birds)	*L fermentum* Treatment, per os (Birds)	*C. jejuni* Treatment, per os (Birds)	Coexposure Treatment, per os (Birds)
0 d	18	18	18	18
1–3 d	18	18 10^9^ CFU/0.2 mL (Probiotic)	18	18 10^9^ CFU/0.2 mL (Probiotic)
4 d	18	18 10^9^ CFU/0.2 mL (Probiotic)	18 10^8^ CFU/0.2 mL (*C. jejuni*)	18 10^9^ CFU/0.2 mL (Probiotic) + 10^8^ CFU/0.2 mL (*C. jejuni*)
5–7 d	18	18 10^9^ CFU/0.2 mL (Probiotic)	18	18 10^9^ CFU/0.2 mL (Probiotic) *per os*
8 d (4 dpi) sample collection	9	9	9	9
9–10 d	9	9	9	9
11 d (7 dpi) sample collection	9	9	9	9

dpi, days post-infection.

**Table 2:** Villus height to crypt ratio in broilers exposed to different bacterial treatments.

Small Intestine Sections	Experimental Groups			
	C	LB	Cj	LBCj
4 dpi				
Duodenum	5.53 ± 0.55	6.20 ± 0.37	5.29 ± 0.37 ^■^	5.68 ± 0.83
Jejunum	5.76 ± 0.47	5.19 ± 0.64	5.80 ± 0.61	5.13 ± 0.63
Ileum	3.04 ± 0.33	3.18 ± 0.23	2.87 ± 0.74	3.35 ± 0.64
7 dpi				
Duodenum	4.40 ± 0.51	5.88 ± 0.60 *	3.95 ± 0.66 ^■^	4.19 ± 0.21 ^■^
Jejunum	5.09 ± 0.44	5.69 ± 1.4	4.96 ± 0.56	4.35 ± 0.37 ^■^
Ileum	2.87 ± 0.34	3.50 ± 0.39*	2.75 ± 0.34 ^■^	3.00 ± 0.16

Values are mean ± SE (*n* = 9). * designates significant differences (Tukey’s test, *p* < 0.05) with the control group; ^■^ with the *L. fermentum* treatment. C, control; LB, *L. fermentum*; Cj, *C. jejuni*; LBCj, coexposure; SE, standard error.

## Data Availability

The data supporting reported results can be found in Table S2. Experimental data.

## Supplementary Materials

The following are available online at https://www.mdpi.com/2076-2615/11/1/235/s1, Table S1. List of primers; Table S2. Experimental data.
