# Supplementary material for: The Probiotic Lactobacillus fermentum Biocenol CCM 7514 Moderates Campylobacter jejuni-Induced Body Weight Impairment by Improving Gut Morphometry and Regulating Cecal Cytokine Abundance in Broiler Chickens
Source: Animals (Basel). 2021 Jan 19;11(1):235. doi: 10.3390/ani11010235 (PMC7832853; doi:10.3390/ani11010235)
Supplement: Supplementary file 1 [file animals-11-00235-s001.zip › animals-1053152-supplementary/TableS1. List of primers- for proofreading.docx]

**Table S1.** List of primers utilized in RT-qPCR for cytokine transcript detection.

| **Primer** | **Sequence 5’-3’** | **Reference** |
| --- | --- | --- |
| IL-1β For  IL-1β Rev | GAAGTGCTTCGTGCTGGAGT ACTGGCATCTGCCCAGTTC | [1] |
| IL-15 For  IL-15 Rev | TGGAGCTGATCAAGACATCTG CATTACAGGTTCCTGGCATTC | [2] |
| IL-17 For  IL-17 Rev | TATCAGCAAACGCTCACTGG  AGTTCACGCACCTGGAATG | [1] |
| IL-18 For  IL-18 Rev | ACGTGGCAGCTTTTGAAGAT  GCGGTGGTTTTGTAACAGTG | [3] |
| IL-4 For  IL-4 Rev | AGCACTGCCACAAGAACCTG  CCTGCTGCCGTGGGACAT | [4] |
| IL-13 For  IL-13 Rev | CATGACCGACTGCAAGAAGGA  CCGTGCAGGCTCTTCAGACT | [5] |
| GAPDH For  GAPDH Rev | CCTGCATCTGCCCATTT  GGCACGCCATCACTATC | [6] |

**References**:

1. Crhanova, M.; Hradecka, H.; Faldynova, M.; Matulova, M.; Havlickova, H.; Sisak, F.; Rychlik; I. Immune response of chicken gut to natural colonization by gut microflora and to *Salmonella enterica* serovar enteritidis infection. *Infect Immun.* **2011**, *79*, 2755–2763.

2. Kolesarova, M.; Spisakova, V.; Matulova, M.; Crhanova, M.; Sisak, F.; Rychlik, I. Characterisation of basal expression of selected cytokines in the liver, spleen, and respiratory, reproductive and intestinal tract of hens. *Vet Med Czech.* **2011**; *56*, 325–332.

3. Šefcová, M.; Levkut, M.; Bobíková, K.; Karaffová, V.; Revajová, V.; Maruščáková, I.C.; Levkutová, M.; Ševčíková, Z.; Herich, R.; Levkut, M. Cytokine response after stimulation of culture cells by zinc and probiotic strain. *Vitr Cell Dev Biol—Anim.* **2019, 55**, 830–837.

4. Truong, A.D.; Park, B.; Ban, J.; Hong, Y.H. The novel chicken interleukin 26 protein is overexpressed in T cells and induces proinflammatory cytokines. *Vet Res.* **2016**, *47*, 65.

5. Cox, C.M.; Sumners, L.H.; Kim, S.; McElroy, A.P.; Bedford, M.R.; Dalloul, R.A. Immune responses to dietary β-glucan in broiler chicks during an Eimeria challenge. *Poult Sci.* *2010*, *89*, 2597–2607.

6. De Boever, S.; Vangestel, C.; De Backer, P.; Croubels, S.; Sys, S.U. Identification and validation of housekeeping genes as internal control for gene expression in an intravenous LPS inflammation model in chickens. *Vet Immunol Immunopathol.* **2008**, *122*, 312–317.
